# Chronic β-Blockade and Systemic Homeostasis: Molecular Integration of Cardiorenal and Immune Pathways, a Narrative Review

**DOI:** 10.3390/biom15121653

**Published:** 2025-11-26

**Authors:** Jason Park, Amethyst Hamanaka, Issac Park, Hosam Gharib Abdelhady

**Affiliations:** 1 College of Osteopathic Medicine, Sam Houston State University, Conroe, TX 77304, USA; afh023@shsu.edu; 2 Department of Intensive Care Unit, National Medical Center, Jung-gu, Seoul 04564, Republic of Korea; wndehrasd@nmc.or.kr

**Keywords:** β-blockers, cardiovascular-renal homeostasis, atherosclerosis and vascular remodeling, hemodynamics, hormonal-immune modulation, genetic polymorphisms, precision medicine

## Abstract

β-blockers (BBs) remain a cornerstone therapy for cardiovascular disorders, reducing heart rate, blood pressure, and arrhythmia risk. Yet, their influence extends well beyond the heart, impacting renal function, inflammatory responses, metabolism, and endocrine balance. Although cardio-selective BBs are designed to minimize off-target effects, they still modulate immune signaling and hormonal pathways, producing paradoxical outcomes. Suppression of sympathetic tone and RAAS activity underpins therapeutic benefit but may also contribute to renal hypoperfusion, electrolyte imbalance, and pro-inflammatory changes, especially in patients receiving combination therapy with RAAS inhibitors or diuretics. Genetic polymorphisms (e.g., ADRB1, GRK5, eNOS, CYP2D6) and comorbidities further shape individual responses. This review integrates cardiovascular, renal, and immune perspectives to map the pathways by which BBs influence systemic homeostasis, highlighting cytokine interactions and disease-specific remodeling. We emphasize the need for personalized, biomarker-guided strategies, leveraging pharmacogenomics, multi-omics, and machine learning tools to optimize BB selection and dosing. By reframing BBs as dynamic modulators of the cardio-renal-immune axis, this review advances their role in precision cardiovascular medicine.

## 1. Introduction

Cardiovascular (CV) disease remains the leading cause of morbidity and mortality worldwide. β-blockers (BBs), particularly cardio-selective agents, improve outcomes in congestive heart failure (CHF), coronary artery disease (CAD), post-myocardial infarction (MI), and arrhythmias by antagonizing β_1_-adrenergic receptors in the heart and kidneys. This blockade reduces cyclic AMP production, decreases Ca^2+^ influx, slows pacemaker depolarization, and suppresses renin release [1,2]. In a retrospective cohort study, BBs were associated with a lower risk of heart failure (HF) and sudden cardiac death (SCD), especially among patients without prior HF [3]. A meta-analysis further confirmed their benefit in HF, including in subgroups with renal impairment, though efficacy declined with worsening renal dysfunction [4].

BBs are now standard therapies in CV disease due to their ability to counter sympathetic drive, lowering heart rate (HR), blood pressure (BP), and myocardial oxygen demand (MOD). However, patient tolerance is strongly shaped by comorbidities such as chronic kidney disease (CKD), necessitating close clinical monitoring [4,5]. Beyond their cardiac actions, BBs reduce renal blood flow (RBF) and glomerular filtration rate (GFR) via β_1_-mediated inhibition of renin release [6,7]. In patients with impaired kidney function, these hemodynamic changes can exacerbate renal hypoperfusion and filtration decline, particularly when autoregulation is already compromised [7].

BB effects also extend to immune remodeling and vascular stability through indirect modulation of angiotensin II (ANG II), nitric oxide (NO), and oxidative stress, largely via suppression of the renin–angiotensin–aldosterone system (RAAS) [7,8]. While β_1_ receptor blockade may influence oxidative stress indirectly through neurohormonal pathways, this relationship remains not fully understood [6]. To maintain systemic homeostasis, compensatory mechanisms engage: the CV system adjusts baroreceptor sensitivity and autonomic tone, while the renal system activates RAAS and alters sodium and fluid handling to preserve blood pressure and intravascular volume [7,8].

This narrative review bridges classical hemodynamic mechanisms with emerging insights into neurohormonal, endocrine, and cytokine signaling, highlighting how these pathways collectively shape BB efficacy and risk. It emphasizes the clinical relevance of genetic polymorphisms, disease heterogeneity, and multi-system interactions in modulating the cardio-renal response to BB therapy. Finally, we propose biomarker-guided, personalized strategies to optimize BB use across overlapping CV and renal comorbidities.

## 2. Methods

This narrative review synthesizes peer-reviewed literature in cardiovascular pharmacology, immunologic signaling, renal physiology, and clinical medicine within a thematic framework suitable for narrative synthesis. Literature was identified through PubMed/MEDLINE and Google Scholar searches covering January 2010 to July 2025, using search terms including “β-blocker,” “cytokines,” “renin,” “aldosterone,” “atherosclerosis and vascular remodeling,” “chronic kidney disease,” “cardiovascular-renal homeostasis,” “hemodynamics,” “hormonal-immune modulation,” “genetic polymorphisms,” “precision medicine” and “hypertension.” Inclusion criteria comprised English full-text reports that provided mechanistic insights or clinically significant information on the physiologic and immunologic effects of BBs. Eligible sources included clinical trials, preclinical research, experimental models, and systematic reviews. Exclusion criteria omitted non-peer-reviewed literature, conference abstracts, case reports, and studies with limited methodological rigor or mechanistic relevance. To provide context for fundamental mechanisms, such as β-receptor selectivity, neurohormonal regulation, and inflammatory signaling, seminal publications predating the primary search window as well as standard pharmacology and cardiovascular training texts were also consulted.

## 3. Synthesis of Literature

This review incorporated 153 references spanning clinical trials, mechanistic studies, pharmacogenetic analyses, and translational reviews addressing the CV and renal effects of BBs. The included literature covered diverse topics such as hemodynamic regulation, cytokine and immune modulation, hormonal signaling, and receptor–genetic interactions, with an emphasis on HF, MI, CKD, and thyrotoxicosis. Because this review followed a narrative rather than a systematic methodology, a PRISMA flowchart was not applied. Instead, Table 1 and Table 2 summarize the key mechanistic and clinical studies, respectively, providing clarity and contextual relevance.

## 4. Disease-Specific Effects of β-Blockers

**Figure 1:**
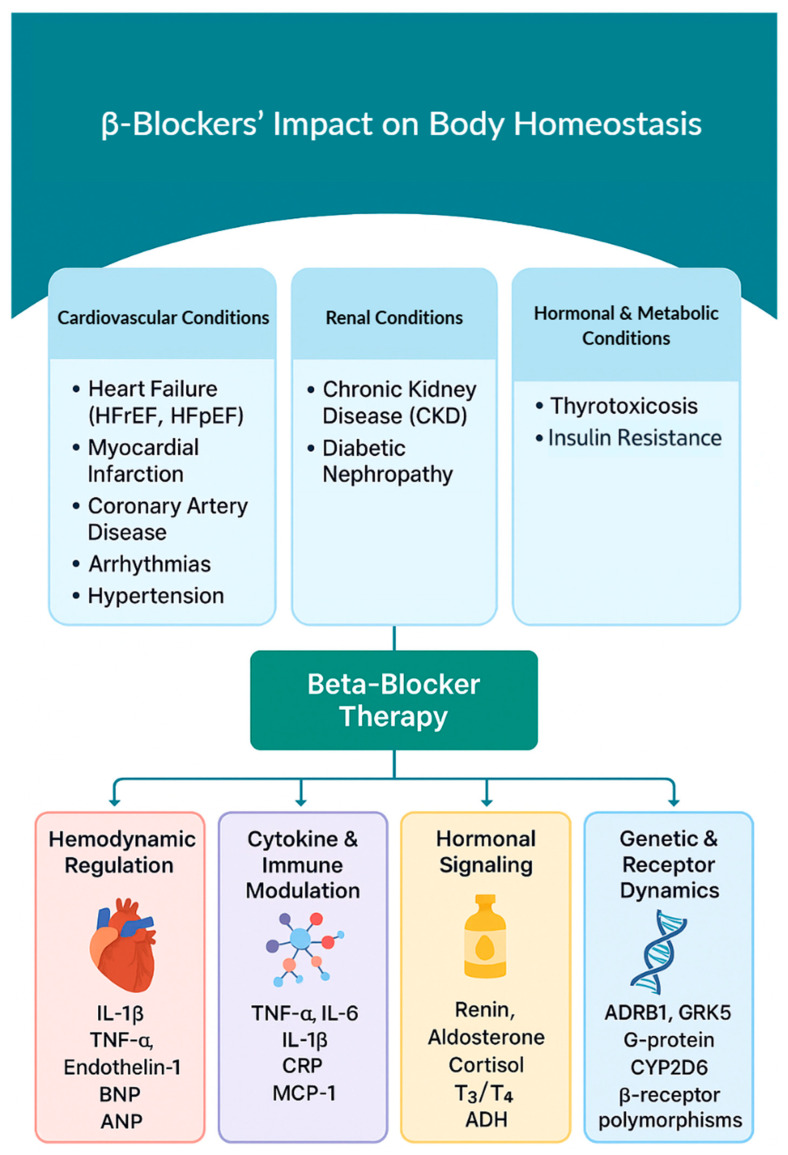
Mechanistic Framework of BBs’ Effects on CV and Renal Homeostasis. The model integrates four key domains: (1) hemodynamic regulation, (2) inflammatory and immune signaling, (3) endocrine regulation, and (4) genetic and receptor-level mechanisms, highlighting representative markers such as IL-1β, IL-6, TNF-α, CRP, renin, aldosterone, BNP, ANP, T_3_/T_4_, and gene polymorphisms (e.g., ADRB1, CYP2D6).

This review organizes the physiologic and molecular effects of BBs into four mechanistic domains: hemodynamics, inflammatory cytokines, hormonal signaling, and genetic mechanisms (Figure 1). This framework offers a versatile lens for understanding how BBs modulate CV and renal homeostasis across diverse clinical settings. Rather than focusing on individual disease states in isolation, this structure emphasizes mechanisms that span multiple pathologies, with disease-specific examples mapped onto these broader domains. For instance, hemodynamic modulation is pivotal in HF and post-MI care, endocrine signaling intersects with thyrotoxicosis and CKD, and genetic polymorphisms influence therapeutic response across conditions. This thematic approach allows clinicians and researchers to identify both shared pathways and disease-specific nuances through which BBs shape disease progression, therapeutic outcomes, and long-term consequences.

### 4.1. β-Blockers in Cardiovascular Disease

#### 4.1.1. Heart Failure (HF)

BBs reduce long-term mortality in chronic HF primarily by mitigating sustained adrenergic overdrive. In acute heart failure (AHF), especially in patients with hemodynamic instability or cardiogenic shock, continuing BB therapy can be unsafe and may trigger severe complications, necessitating an individualized risk–benefit assessment [39]. Registry data support this cautious approach: in the Kyoto Congestive Heart Failure (KCHF) study of 3817 patients with acute decompensated HF (ADHF), BB use at admission was independently associated with lower in-hospital mortality, particularly among patients with prior HF hospitalizations [27].

In HF with reduced ejection fraction (HFrEF), BBs consistently improve survival through suppression of sympathetic hyperactivity, reduction in MOD, and enhancement of left ventricular ejection fraction (LVEF) [40]. In contrast, in HF with preserved ejection fraction (HFpEF), characterized by diastolic dysfunction and lower neurohormonal activation, randomized trials have not demonstrated prognostic benefit from BB therapy [28]. Patients with HF with mildly reduced ejection fraction (HFmrEF, 41–49%) may exhibit enhanced RAAS and SNS activation and thus appear more responsive to neurohormonal therapies, including BBs, although robust, randomized evidence is lacking [41].

Pharmacogenomics strongly influences the BB response in HF. The ADRB1 Arg389Gly variant alters receptor sensitivity; Arg389 homozygotes demonstrate greater BB responsiveness, improved LVEF, and enhanced survival in HFrEF, though not consistently in acute coronary syndrome (ACS) populations [9,10,11,12]. The GRK5 Leu41 allele, more common in African American patients, produces a “genetic β-blockade” via enhanced receptor desensitization and is associated with improved outcomes [12]. Beyond desensitization, GRK5 phosphorylates histone deacetylase 5 (HDAC5), reversing maladaptive Gαq-mediated transcription and promoting adaptive myocardial remodeling [10]. Other polymorphisms, including ADRA2C, GRK4, and ADRB2 variants (Gly16Arg, Glu27Gln), are also under investigation as biomarkers of BB efficacy and tolerability, underscoring the potential for genotype-guided therapy [9,10,11,12]. Beyond genetic determinants, immunomodulation represents an emerging therapeutic axis. BBs such as carvedilol and bisoprolol inhibit pro-inflammatory cytokines, including tumor necrosis factor-α (TNF-α) and interleukin-6 (IL-6), disrupt the cytokine–reactive oxygen species (ROS) amplification cycle, and restore endothelial nitric oxide synthase (eNOS) homeostasis. These findings position BBs not only as hemodynamic modulators but also as potential regulators of inflammatory remodeling in HF [42].

#### 4.1.2. Post-Myocardial Infarction (MI)

Post-MI therapy with BBs attenuates SNS hyperactivity and stabilizes cardiac electrical conduction via β-adrenergic receptor blockade, resulting in significant reductions in mortality, reinfarction, and ventricular arrhythmias [32,33,34,43]. Through selective inhibition of myocardial β_1_-receptors, BBs reduce HR, myocardial contractility, and BP, thereby lowering MOD and limiting infarct expansion [44,45]. Recent findings suggest that BBs can modulate immune-inflammatory responses following MI by suppressing pro-inflammatory cytokines such as TNF-α and IL-6, decreasing leukocyte recruitment, limiting infarct-related remodeling, and maintaining myocardial tissue [46]. However, chronic β-receptor blockade can impair hepatic glycogenolysis and gluconeogenesis, increasing the risk of hypoglycemia, particularly in diabetic patients [33,44,46]. Thus, clinicians must balance the survival benefits of BB therapy against potential metabolic hazards, with vigilant glucose monitoring recommended to optimize outcomes [34,45]. Genetic polymorphisms, including PAI-1 (4G/5G), MMP-3 (5A/6A), IL-18, LDLR, APOA5, LTA, LGALS2, AT2 (−1332 G/A), ALDH2 (RS671), CX37, AKAP12, and GLRA2, have been associated with MI risk through pathways involving fibrinolysis, lipid metabolism, vascular inflammation, endothelial function, and contractility [47]. While the clinical application of pharmacogenetics to BB therapy remains investigational, β_1_-selective agents such as metoprolol and bisoprolol show promise by reducing sympathetic activation, suppressing inflammatory cytokine activity, and inhibiting renin release in genetically susceptible populations [46,47].

#### 4.1.3. Acute and Chronic Coronary Diseases

In ACS, BBs are primarily used for symptom relief and reduction in arrhythmias and ischemia [48]. Current guidelines recommend early oral administration within 24 h of presentation when no contraindications exist, whereas routine early intravenous use is discouraged in patients at risk of cardiogenic shock [48]. In chronic CAD, BBs reduce HR, myocardial contractility, and arterial pressure, thereby lowering MOD and improving anginal symptoms [49]. However, routine long-term use for outcome improvement is not generally supported unless another indication exists, such as recent MI, LVEF ≤ 50%, or arrhythmia; thus, therapy should be individualized [49]. Potential adverse effects of prolonged treatment include fatigue and sexual dysfunction, particularly in younger patients, possibly related to endothelial dysfunction and diminished NO bioavailability [50].

Across both ACS and stable CAD, BBs restore supply-demand balance and can improve outcomes, though long-term use may be associated with adverse CNS or metabolic effects [48,49,50]. Pro-inflammatory cytokines, including IL-12p70 and IL-17, are independently associated with CAD severity [51], suggesting a potential immunomodulatory role of BBs beyond hemodynamic effects. Given that CAD itself may produce ECG abnormalities such as poor R-wave progression [52], and BBs can cause bradycardia or atrioventricular block, careful ECG and HR monitoring during initiation and titration is essential [53].

Supporting selective use, a large new-user cohort study of 28,039 patients ≥ 66 years old with angiographically confirmed obstructive CAD (without recent MI or HF) demonstrated that BB initiation within 90 days of angiography was associated with a modest but significant reduction in the 5-year composite outcome of all-cause death, MI, or HF hospitalization (HR 0.92; 95% CI 0.86–0.98; *p* = 0.006), driven primarily by a reduction in MI hospitalizations (HR 0.87; 95% CI 0.77–0.99), with no effect on overall mortality or HF outcomes [29]. Finally, genetic factors may influence CAD pathogenesis and BB responsiveness. TGFβ_1_ and its receptor polymorphisms have been linked to vascular inflammation and extracellular matrix (ECM) remodeling [54]. In addition, ADRB1 (Arg389Gly) and ADRB2 (Gly16Arg, Glu27Gln) variants have been associated with altered β-adrenergic signaling and treatment efficacy, reinforcing the potential role of pharmacogenetics in tailoring BB therapy [9,10,11,12].

#### 4.1.4. Arrhythmias

**Figure 2:**
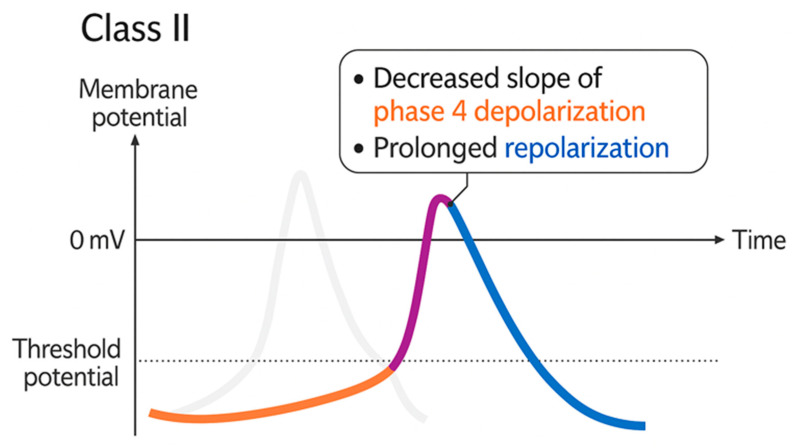
Electrophysiological Effects of Class II BBs on Pacemaker Action Potential. The drugs reduce the slope of phase 4 depolarization in pacemaker cells, slowing conduction through the atrioventricular node, and prolonging the effective refractory period, which collectively reduces heart rate and suppresses arrhythmias [56].

In hereditary arrhythmogenic disorders, such as catecholaminergic polymorphic ventricular tachycardia (CPVT), BBs, particularly propranolol, are a cornerstone therapy for preventing sudden cardiac death (SCD) by reducing catecholamine-induced excitability and suppressing ventricular arrhythmias (Figure 2) [55]. According to the Vaughan Williams classification, antiarrhythmic drugs are categorized into four main classes based on their mechanisms of action: Class I (fast sodium channel blockers), Class II (β-blockers), Class III (potassium channel blockers), and Class IV (calcium channel blockers). As Class II agents, BBs act primarily by reducing the slope of phase 4 depolarization in pacemaker cells, slowing conduction through the atrioventricular node, and prolonging the effective refractory period, thereby reducing HR and suppressing arrhythmias [56]. In other contexts, such as during electroconvulsive therapy (ECT), BBs stabilize hemodynamics by blunting HR peaks with minimal additional adverse effects [57]. Beyond their electrophysiologic actions, certain BBs may exert pleiotropic effects on vascular and inflammatory processes, though the clinical relevance of these effects for arrhythmia prevention remains under investigation [1]. BB pharmacokinetics are influenced by genetic polymorphisms. For example, CYP2D6 variation alters metoprolol exposure: poor metabolizers (PMs) exhibit elevated plasma concentrations and increased risk of bradycardia, whereas ultra-rapid metabolizers (UMs) may require alternative dosing strategies due to reduced efficacy [58]. These findings support a personalized approach to BB therapy, including careful dose titration and, when feasible, genotype-guided selection to maximize efficacy and safety in CV management, particularly in genetically vulnerable populations.

### 4.2. β-Blockers and Renal System Dysfunction

#### 4.2.1. Chronic Kidney Disease (CKD)

In patients with CKD, BBs help reduce cardiac load and may lower the markedly elevated risk of SCD, which can be up to 50 times higher in end-stage renal disease compared with that in the general population [31]. BBs suppress sympathetic overactivity, reduce afterload, and normalize BP, improving cortical and medullary oxygenation and potentially offering renal protection, though long-term benefits remain uncertain [59]. CKD is characterized by chronic inflammation, oxidative stress, and RAAS hyperactivation. While direct evidence for cytokine modulation is limited, BBs may exert anti-inflammatory effects through sympathetic-immune regulation [31]. By inhibiting renin secretion, BBs can counteract RAAS hyperactivation. When combined with RAAS inhibitors, BBs may provide better BP control with reduced volume-mediated nephrotoxicity [31]. Evidence from meta-analyses indicates that BB therapy in CKD patients with systolic HF is associated with a 28% relative reduction in all-cause mortality (RR 0.72) and a 34% relative reduction in cardiovascular mortality (RR 0.66). However, the therapy increases the risk of bradycardia and hypotension, especially in patients on dialysis [35]. While BBs are not considered first-line therapy in CKD patients without HF, vasodilating BBs such as carvedilol, an agent that block α_1_, β_1_, and β_2_ adrenergic receptors, offer renal-protective and metabolic benefits, making them reasonable second- or third-line therapeutic options [31,36]. Pharmacogenomic variability may influence BB efficacy and safety in CKD. Polymorphisms in ADRB1 (Arg389Gly), ADRB2 (Gly16Arg, Glu27Gln), CYP2D6 metabolism, and eNOS (NOS3) variants (Glu298Asp, –786T>C) may alter therapeutic response, vascular protection, and risk of adverse effects, particularly in patients with CV comorbidities or those undergoing dialysis [9,10,11,12,60,61].

#### 4.2.2. Diabetic Nephropathy (DN)

Response to BBs in DN may be influenced by patient-specific genetic factors that intersect with hemodynamic, inflammatory, and oxidative stress pathways. Polymorphisms in genes such as ACE, IL-6, TNF-α, COL4A1, eNOS, SOD2, APOE, and GLUT1 have been implicated in DN progression, highlighting the role of genetic determinants in disease pathophysiology [62]. For example, ACE I/D variants modulate RAAS activity, a pathway indirectly inhibited by BBs through sympathetic suppression [62]. BBs may also modulate chronic low-grade inflammation by intersecting with TNF-α and IL-6 signaling, while polymorphisms in eNOS and SOD2 influence NO bioavailability and oxidative stress. Antioxidant BBs, such as carvedilol and nebivolol, may confer renal protection under these conditions [62,63]. Metabolic regulation is also relevant: impaired GLUT1/GLUT4 expression, as observed in diabetic cardiomyopathy (DCM), affects glucose transport and cellular energy handling. BBs may indirectly improve this axis by lowering myocardial and renal metabolic demands and enhancing insulin sensitivity [63]. Furthermore, dysregulation of matrix metalloproteinase-2 (MMP-2), a key mediator of extracellular matrix remodeling in both DCM and DN, may be modulated by BB therapy, as evidenced by alterations in circulating MMP-2/TIMP-2 levels in CKD patients [61,63]. Collectively, these findings support a precision medicine approach in DN, wherein BB therapy could be tailored based on receptor profiling and genetic testing to optimize renal and cardiovascular outcomes.

### 4.3. Hormonal and Metabolic Effects of β-Blockers

Long-term BB therapy modulates the SNS and hypothalamic–pituitary–adrenal (HPA) axis, influencing adaptation to stress, hormonal homeostasis, and metabolic control over time [30,64]. Clinical and exercise studies indicate that both nonselective and β_1_-selective BBs significantly alter neurohormonal responses, namely cortisol, E, and prolactin during physical stress, but not baseline levels of hormones [65]. These alterations may blunt appropriate stress-induced hormonal peaks, potentially impairing physiological adaptation in the context of acute or chronic stress [30,64]. For example, in hypertensive men, propranolol or metoprolol infusion during exercise increased lactate levels and decreased free fatty acid mobilization, demonstrating BBs’ impact on energy substrate regulation under adrenergic stimulation [30]. BBs reduce cortisol release by inhibiting β-adrenergic stimulation of adrenal responsiveness to CRH and ACTH [66,67]. This mechanism has been reported to be anecdotally effective in subclinical cases of ACTH-independent macronodular adrenal hyperplasia (AIMAH) associated with Cushing’s syndrome, as demonstrated by a case in which propranolol normalized serum and urinary cortisol levels by blocking ectopically expressed β-adrenergic receptors in the adrenal cortex [68]. However, in the general population, chronic cortisol suppression has been suggested to compromise stress resilience, particularly among psychiatric or surgical patients [66,67]. BBs also influence thyroid hormone metabolism. Nonselective BBs such as propranolol inhibit 5′-deiodinase and hence decrease peripheral conversion of T_4_ to T_3_ and thereby decrease active thyroid hormone levels and alleviate the symptoms of hyperthyroidism, including tachycardia, tremor, and widened pulse pressure [37,38]. Propranolol is therefore useful in acute management of thyrotoxicosis. Conversely, in euthyroid or hypothyroid patients, propranolol may exacerbate fatigue, cold intolerance, or depression by further lowering T_3_ availability, underscoring the importance of monitoring thyroid function in long-term BB therapy [38].

Metabolically, BBs can impair glucose tolerance and increase insulin resistance. Vasodilatory BBs such as carvedilol appear to preserve insulin-mediated endothelial function and may be a better alternative in diabetic patients [69,70]. For example, metoprolol attenuates insulin-stimulated vasodilation, whereas carvedilol preserves endothelial responsiveness, demonstrating subclass-specific effects on metabolism [70]. BBs also suppress plasma renin activity, contributing to their antihypertensive efficacy. When combined with drugs that act on the RAAS, e.g., ACE inhibitors or diuretics; however, this suppression may increase the risk of hyperkalemia (especially in CKD), whereas hyponatremia is primarily associated with thiazide diuretics [71]. In conditions of mineralocorticoid excess or sodium retention, BBs can also alter renal hemodynamics and fluid balance. Therefore, individualized therapy is needed, particularly in comorbid patients [72]. Table 3 summarizes the key hormonal and metabolic effects associated with long-term BB therapy and provides a practical framework for clinical application.

## 5. Biochemical Pathways and Cytokine Interactions in BBs Therapy

All three β-adrenergic receptor subtypes (β_1_, β_2_, β_3_) primarily couple to Gs proteins, leading to adenylyl cyclase activation, increased cAMP, and downstream PKA-mediated effects, with tissue-specific expression and target proteins contributing to functional variation [73].

### 5.1. β-Adrenergic Receptor Interactions

#### 5.1.1. β_1_ Receptor (Cardiac & Renal)

BBs reduce BP and improve vascular function primarily through negative chronotropic and inotropic effects, as well as the inhibition of renin release [73]. In ANGII-infused rabbit models, β_1_-adrenergic receptor stimulation is protective against NE-induced vasoconstriction and oxidative stress in renal afferent arterioles via cAMP-mediated signaling, which can be reversed by the β_1_-selective antagonist CGP-20,712A [7]. Certain BBs with intrinsic sympathomimetic activity (ISA), such as carteolol and alprenolol, possess partial agonist activity at β_2_-adrenergic receptors. Although experimental models show carteolol increases systolic BP, HR, and cAMP signaling in a dose-dependent manner independent of β_1_ blockade [13,14,45], in clinical practice ISA agents primarily attenuate bradycardia and preserve exercise HR without causing sustained BP elevation. They provide reduced survival benefit in HF due to reduced β-blockade but can be therapeutically useful in active hypertensives by maintaining vascular tone and CO while avoiding marked bradycardia.

Subclasses differ as carvedilol and labetalol provide additional vasodilation through α_1_-adrenergic receptor blockade, while nebivolol enhances endothelial NO bioavailability [74]. BBs also decrease HR and MOD, enhancing diastolic filling time and thereby improving coronary perfusion. These anti-ischemic effects make BBs particularly useful in stable angina and ischemic heart disease (IHD), where they counter excessive β-adrenergic stimulation by NE and E [74]. These mechanisms, along with subtype-specific actions, underscore their efficacy in HTN, ischemic disease, and HF [74,75]. By suppressing chronic sympathetic overactivity, BBs also facilitate reverse cardiac remodeling, improve systolic and diastolic left ventricular function, reduce preload and afterload, and suppress malignant arrhythmias. These synergistic actions alleviate anginal symptoms, lower MI risk, and improve long-term survival in patients with HF and IHD [75]. Furthermore, BBs stabilize cardiac electrophysiology through their negative chronotropic, dromotropic, and inotropic effects, making them first-line agents for ventricular rate control in atrial fibrillation and for preventing life-threatening arrhythmias. In congenital syndromes such as long QT syndrome and CPVT, BBs such as nadolol reduce syncope and SCD by inhibiting adrenergic-induced, calcium-dependent arrhythmogenic triggers [1,26,76].

In renal physiology, β_1_-adrenergic receptors on juxtaglomerular (JG) cells regulate renin release via SNS input-activated cAMP-dependent pathways. BBs inhibit this β_1_-mediated pathway and thereby inhibit renin release, dampen catecholamine effects, and downregulate the RAAS [15,16,77]. β_2_-receptor blockade in renal tubules has also been proposed to reduce sodium reabsorption, although the primary renal action of BBs remains β_1_-mediated inhibition of renin release. These actions necessitate close monitoring of renal function, especially with metabolic diseases such as diabetes, where RAAS dysregulation and renal injury are common [78,79]. Despite potential reductions in renal perfusion, BBs reduce oxygen demand in renal tissue, thereby supporting hemodynamic stability and mitigating ischemic stress in hypertension (HTN) and renovascular disease [59,80]. Table 4 illustrates the mechanisms by which β_1_-selective BBs influence cardiac and renal physiology.

#### 5.1.2. β_2_ Receptor (Vascular, Pulmonary, Metabolic)

β_2_-adrenergic receptors are expressed predominantly on bronchial smooth muscle, vascular endothelium, liver, pancreas, and skeletal muscle. Activation of β_2_-receptors normally induces bronchodilation, vasodilation, and metabolic regulation via Gs protein–coupled cAMP/PKA signaling [81]. Nonselective BBs, such as propranolol, inhibit both β_1_ and β_2_ receptors, producing clinically relevant effects by reducing bronchodilation, increasing vascular tone, and disrupting glucose homeostasis.

Loss of β_2_-mediated bronchodilation can cause bronchoconstriction and a reduction in FEV_1_, posing a particular risk in patients with asthma or COPD due to heightened airway responsiveness [82]. Additionally, β_2_-receptor blockade in the liver and skeletal muscle may reduce glycogenolysis and insulin-mediated glucose uptake, increasing the risk of hypoglycemia or impaired exercise tolerance, especially in diabetic or insulin-resistant patients [81,82].

**Figure 3:**
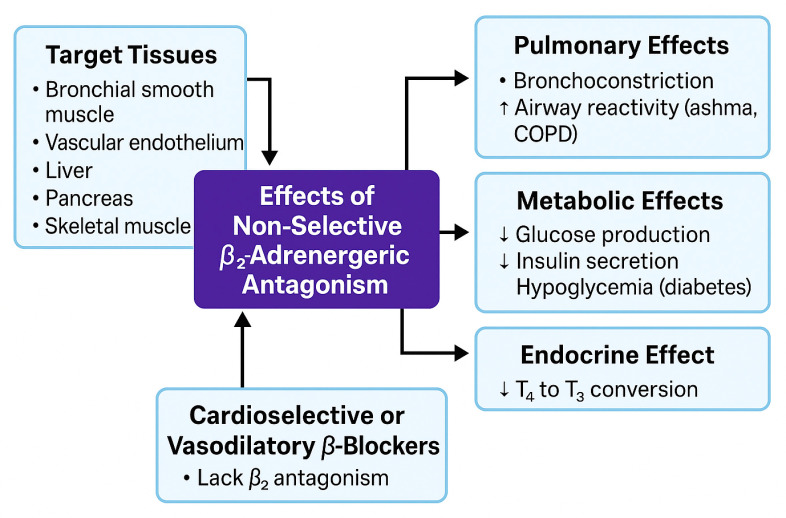
The Systemic Effects Associated with Nonselective β_2_-adrenergic Receptor Blockade. This figure illustrates the pulmonary, vascular, metabolic, and endocrine effects resulting from nonselective β_2_-receptor blockade.5.1.3. β_3_ Receptor (Adipose Tissue, Metabolism).

Metabolically, β2-antagonism reduces hepatic glucose production by inhibiting glycogenolysis and gluconeogenesis, suppresses insulin release from pancreatic β-cells, increases circulating glucose levels, and masks adrenergic warning symptoms, such as tachycardia. These effects heighten the risk of hypoglycemia unawareness in insulin-treated patients and may worsen insulin resistance in individuals with visceral obesity or metabolic syndrome [83,84]. Cardioselective BBs (e.g., metoprolol, bisoprolol) largely spare β_2_ receptors and are therefore preferred in patients at risk of bronchospasm or metabolic complications. Vasodilating BBs, such as carvedilol, despite some β_2_ antagonism, functionally act as β_2_-sparing agents due to their α1-blockade and NO-mediated endothelial vasodilation. These agents enhance insulin sensitivity and provide metabolic benefits [70]. Propranolol also inhibits type I 5′-deiodinase, decreasing peripheral conversion of thyroxine (T_4_) to triiodothyronine (T_3_) [85]. This is beneficial in thyrotoxicosis by alleviating symptoms such as tachycardia and tremors but may exacerbate hypothyroid states with prolonged or excessive use. Figure 3 summarizes the systemic effects associated with nonselective β_2_-adrenergic receptor blockade, integrating pulmonary, vascular, metabolic, and endocrine consequences.

β_3_-adrenergic receptors, although less commonly discussed, play important roles in adipose tissue, brown adipose tissue, and the urinary bladder, mediating lipolysis, thermogenesis, and detrusor muscle relaxation. Third-generation BBs, such as carvedilol and nebivolol, do not directly target β_3_ receptors but indirectly modulate β_3_-associated NO signaling pathways, enhancing endothelial function and promoting vasodilation, which contributes to improved CV [86]. These agents also modulate adipokine profiles by upregulating adiponectin and decreasing leptin, a shift that may improve insulin sensitivity, regulate appetite, and reduce long-term cardiometabolic risk. This is particularly relevant in patients with type 2 diabetes or obesity, where leptin dysfunction contributes to endothelial impairment and increased CV risk [87].

**Figure 4:**
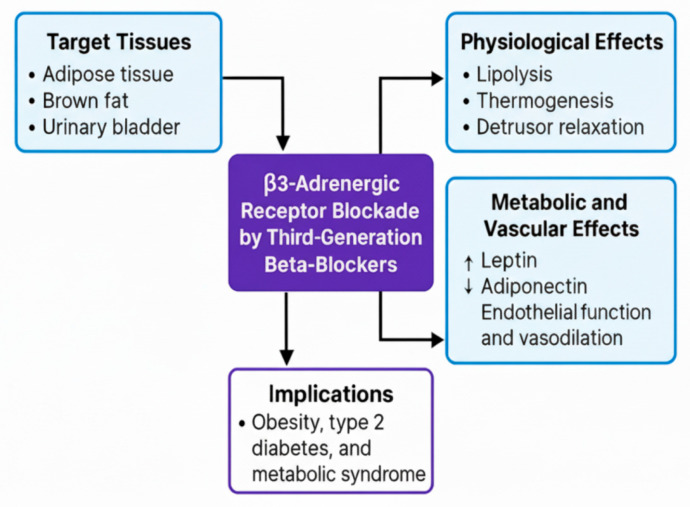
β_3_-adrenergic Receptor Modulation Across Physiological, Metabolic, and Clinical Contexts. This figure depicts the key physiological, metabolic, and clinical effects of β_3_-receptor modulation by third-generation β-blockers.

Thus, third-generation BBs exert an indirect effect on β_3_-adrenergic signaling, underscoring their broader metabolic and vascular impact. This is particularly relevant in patients with obesity, type 2 diabetes, or metabolic syndrome, where dysregulated leptin pathways and impaired β_3_ signaling may influence clinical outcomes. Figure 4 illustrates the physiological, metabolic, and clinical effects of β_3_ modulation by third-generation BBs in CV and metabolic contexts [86,87].

## 6. Discussion

As discussed above, BBs remain a cornerstone in the treatment of CV conditions. However, their systemic influence extends far beyond the CV system, engaging a complex network of homeostatic, neurohormonal, immunologic, and renal pathways. This discussion integrates the interconnected physiological domains, particularly the RAAS, cytokine networks, NO signaling, and macrophage polarization to illustrate both the therapeutic benefits and potential drawbacks of long-term BB use, with renal involvement serving as a hallmark of chronic hemodynamic remodeling.

### 6.1. RAAS and Immune Modulation

BBs inhibit renin release by blocking β_1_-adrenergic stimulation of JG cells, thereby suppressing RAAS activation and downstream ANG II production [15,16]. RAAS inhibition provides CV and renal protection through BP lowering and antifibrotic effects, especially in HF and CKD [31,36]. ANG II promotes pro-inflammatory signaling via AT_1_ receptor (AT_1_R)-mediated activation of protein kinase C (PKC), STAT1, and NF-κB, which increases IL-6 secretion and cyclooxygenase-2 (COX-2) expression [17,88,89]. These cascades drive vascular remodeling, renal injury, and chronic inflammation. By contrast, AT_2_ receptor (AT_2_R) activation exerts anti-inflammatory effects through upregulation of PPARγ and suppression of NF-κB activity, thereby reducing IL-6 signaling [90]. COX-2 amplifies inflammation by generating prostaglandin E_2_ (PGE_2_). While PGE_2_ induces vasodilation via EP2/EP4 receptors, it paradoxically suppresses inducible NO synthase (iNOS) expression, lowering NO availability in immune cells. This dual role diminishes NO-mediated immune regulation in chronic inflammatory states [91,92,93]. In DN, hyperglycemia-induced COX-2 overexpression in podocytes contributes to proteinuria, mesangial expansion, and podocyte foot process effacement, changes that are attenuated by RAAS inhibition or COX-2 blockade [91,92,93]. By limiting ANG II generation, BBs indirectly modulate this axis by suppressing AT_1_R-driven COX-2 expression, PGE_2_ release, and downstream pro-inflammatory signaling. This restores immune balance via the COX-2–PGE_2_–iNOS pathway, reduces chronic inflammation, and preserves renal function, particularly in patients with metabolic or inflammatory comorbidities [31,36,91,92,93].

Aldosterone and antidiuretic hormone (ADH), both downstream of RAAS, also contribute to fluid and electrolyte homeostasis. Their activity is indirectly regulated by BB-mediated RAAS suppression [94]. While COX-2 does not directly control renin release, it mediates many ANG II-dependent vascular and immune responses [16,95]. Thus, by inhibiting β_1_-mediated renin release, BBs curb ANG II formation, blunt AT_1_R-driven inflammatory cascades, and mitigate COX-2/PGE_2_ dysregulation (Table 5). This integrated mechanism underpins their cardio-renal protective and immunomodulatory benefits [17,96].

**Figure 5:**
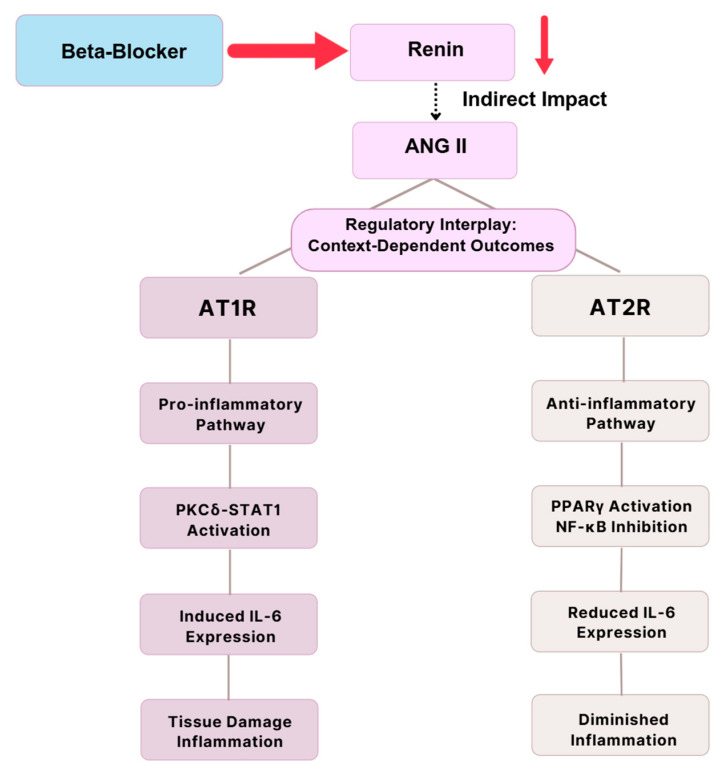
ANGII Modulation of IL-6 via AT_1_R and AT_2_R Pathways, adapted from prior studies. AT1R-driven signaling via PKC, STAT1, and NF-κB promotes IL-6 and COX-2 expression, whereas AT2R-mediated PPARγ activation and NF-κB suppression counteract inflammation. Although current evidence highlights these opposing mechanisms, further mechanistic mapping and disease-specific studies are needed to fully delineate these pathways [17,88,90,91,92,95,96,98,99].

However, ANG II adds complexity to IL-6 modulation through its interaction with AT_1_R and AT_2_R. AT_1_R promotes PKCδ, STAT1, and other pro-inflammatory pathways and induces IL-6 production, leading to tissue damage and chronic inflammation [97,98]. Alternately, AT_2_R signaling elicits anti-inflammatory responses through the enhancement of peroxisome PPARγ activation or inhibition of NF-κB activity, which decreases IL-6 expression and reduces inflammation [90,99]. This balance demonstrates the context-dependent nature of ANG II effects on inflammatory responses via AT_1_R or AT_2_R signaling, as shown in Figure 5.

In addition to the AT_1_R- and AT_2_R-mediated cytokine signaling outlined above, systemic vascular tone also depends upon a balance between vasoconstrictors and vasodilators. ANG II, ACTING through AT_1_R, remains one of the most potent vasoconstrictors, both raising vascular resistance and inducing COX-2 expression with downstream generation of prostaglandins such as PGE_2_ [17,88,89,95]. This convergence of mechanical stress and inflammatory signaling synergistically enhances fibrosis and endothelial dysfunction. Counter-regulatory mediators such as NO, prostacyclin (PGI_2_), and bradykinin oppose these effects by inducing vasodilation and, in some cases, attenuating COX-2-mediated pathways [91,92,93]. BBs suppress renin secretion and thereby diminish ANG II-mediated vasoconstrictive and COX-2-inducing impacts [15,16,80,96,100], while RAAS inhibitors act more directly at the receptor or enzymatic level [31,36]. Together, these mechanisms of therapy shift the hemodynamic and immunologic balance away from ANG II-induced vasoconstriction and inflammation to vasodilation, vascular protection, and preservation of organ function in the long term [80,90,96,100].

### 6.2. Nitric Oxide and Vascular Homeostasis

BB-mediated inhibition of RAAS signaling indirectly suppresses downstream effectors such as COX-2 and PGE_2_, thereby modulating NO bioavailability, a central determinant of vascular tone, renal perfusion, fluid-electrolyte balance, and immune homeostasis. NO is generated by three NOS isoforms: endothelial NOS (eNOS), neuronal NOS (nNOS), and inducible NOS (iNOS), each activated under distinct physiological or pathological conditions [18].

BBs-mediated inhibition of RAAS signaling indirectly suppresses downstream effectors such as COX-2 and PGE_2_, thereby modulating NO bioavailability, a critical regulator of vascular tone, renal perfusion, fluid-electrolyte balance, and immune homeostasis. NO is produced by three NOS isoforms: eNOS, neuronal NO synthase (nNOS), and inducible iNOS, each regulated under distinct physiological or pathological conditions [18]. In inflammatory and immunological settings, PGE_2_ modulates iNOS expression via interferon regulatory factor 1 (IRF1), and prolonged suppression of COX-2/PGE_2_ during BB therapy may reduce iNOS-derived NO [101,102,103]. This can lead to NO deficiency, promoting vascular dysfunction, platelet activation, and cytokine imbalance, particularly in patients with diabetes or CKD [104]. Beyond immunity, nNOS-derived NO contributes to neurohormonal regulation by influencing vasopressin, oxytocin, and atrial natriuretic peptide (ANP) release through COX-2 and adenosine pathways [105,106,107,108,109,110]. BB-induced suppression of ANG II and RAAS activity may blunt this signaling, predisposing patients to hyponatremia, fluid overload, or impaired hormonal feedback, especially when combined with diuretics or ACE inhibitors [107,110,111]. In the kidney, NO maintains afferent arteriolar vasodilation, GFR, and tubuloglomerular balance. Excessive RAAS blockade or reduced iNOS activity may impair these mechanisms, causing renal hypoperfusion, vasoconstriction, and ischemic tubular injury. The risk is heightened with the NSAID use, dehydration, or polypharmacy involving RAAS inhibitors and diuretics [112]. Furthermore, reduced GFR has been linked with an expansion of terminally differentiated T cells (e.g., CD11a^++^CD28^−^CD57^+^), which impair immune resolution and tissue repair [113,114,115]. Conversely, third-generation BBs such as Nebivolol exert favorable NO-related effects. By stimulating eNOS, activating AMP-activated protein kinase (AMPK), and promoting GLUT4 translocation, Nebivolol enhances endothelial function, induces vasodilation, and improves insulin sensitivity in patients with diabetes or metabolic syndrome [79,116,117,118]. Nebivolol also downregulates pathological iNOS activity, preserving protective NO signaling without exacerbating oxidative stress [19].

**Figure 6:**
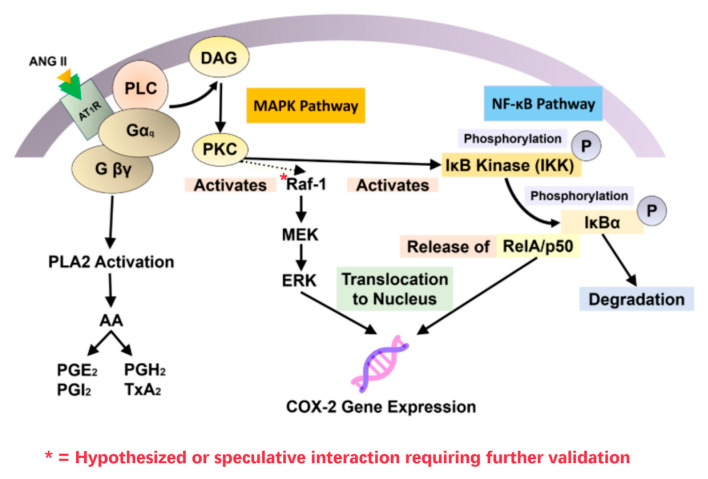
Angiotensin II–mediated signaling pathways regulating COX-2 expression. Binding of ANG II to AT1R activates the mitogen-activated protein kinase (MAPK) and NF-κB pathways, as well as phospholipase A2 (PLA2). Crosstalk between PKC and MAPK enables PKC to activate Raf-1 and downstream ERK, leading to ERK-mediated transcriptional regulation of the COX-2 gene [79,116]. In parallel, PKC activates IκB kinase (IKK) within the NF-κB pathway, further promoting COX-2 expression [117].

Together, these findings highlight the therapeutic duality of BB-induced NO modulation: depending on the agent, dose, and comorbid context, effects can either impair vascular-renal balance or enhance cardiometabolic outcomes. Figure 6 illustrates these interactions across RAAS suppression, COX-2/PGE_2_ pathways, NOS isoforms, and their vascular, renal, and immune consequences.

### 6.3. Immune Remodeling and Macrophage Polarization

Persistent ANG II signaling remodels the vasculature through both structural and immunologic mechanisms. Structurally, AT_1_R activation induces smooth muscle hypertrophy and extracellular matrix (ECM) accumulation via profibrotic pathways, including TGF-β, MAPK, and MMP cascades, leading to arterial fibrosis and stiffening [17,88,95,119,120]. Immunologically, ANG II skews macrophage polarization toward the pro-inflammatory M1 phenotype. These M1 macrophages release cytokines such as TNF-α, IL-6, and IL-1β, thereby amplifying vascular inflammation and renal injury [120,121]. In contrast, AT_2_R activation promotes M2 macrophage differentiation, which supports tissue repair and fibrosis resolution through PPARγ activation and NF-κB modulation [90,99]. Clinically, these signaling programs translate into distinct cardiac outcomes. Pressure overload drives concentric hypertrophy and fibrosis, volume overload induces eccentric dilation, and post-MI recovery hinges on a timely transition from M1 to M2 macrophages to stabilize scar formation. BBs, particularly when combined with ACE inhibitors, facilitate this reparative shift by lowering wall stress, suppressing inflammation, and preserving structural integrity [122,123,124,125]. Atherosclerosis further illustrates maladaptive immune remodeling. ANG II enhances monocyte adhesion to activated endothelium, promotes macrophage differentiation, and increases ROS production [126,127]. These processes drive endothelial dysfunction, smooth muscle migration, and ECM deposition, forming fibrous caps that can be destabilized by MMP overactivity, precipitating rupture and acute coronary syndromes [128,129]. Ultimately, the balance between M1 and M2 macrophages dictates whether vascular remodeling culminates in progressive damage or reparative healing [130,131].

#### 6.3.1. M1 Pro-Inflammatory Signaling

BBs modulate immune dynamics indirectly through neurohormonal and renal mechanisms. By suppressing renin release, they attenuate RAAS-mediated sodium retention and vasoconstriction, thereby contributing to blood pressure control and renal protection [132,133,134,135]. These renal effects extend into immunomodulation, particularly in shaping macrophage polarization. In CKD and related disease states, macrophages are preferentially skewed toward the pro-inflammatory M1 phenotype. Clinical and translational studies support this association, showing that systemic inflammation, characterized by elevated TNF-α, IL-6, and IL-10, along with altered immune checkpoints such as PD-1, is highly prevalent in HF and atrial fibrillation, reinforcing the role of immune dysregulation in cardio-renal syndromes [24,136]. At the molecular level, pathogenic processes are amplified by intracellular signaling cascades including Notch, NF-κB, JAK-STAT, and PI3K-AKT, which sustain inflammatory activation and propagate tissue injury [137,138,139,140]. Collectively, these cytokines and signaling pathways constitute central therapeutic targets for limiting immune-mediated kidney injury and the broader spectrum of cardio-renal syndromes.

#### 6.3.2. M2 Reparative and Fibrotic Roles

In contrast to M1-mediated inflammation, immune resolution and tissue repair are associated with M2 macrophage activities. Polarized by IL-4, IL-10, or IL-13, M2 macrophages orchestrate anti-inflammatory responses and fibrotic signaling through TGF-β_1_/Smad3, with NLRP3 inflammasome activation in certain contexts [25]. While protective in acute tissue injury, chronic M2 activation may contribute to maladaptive fibrotic remodeling. Thus, the M2 macrophage repair-fibrosis function is highly context-dependent and relies on temporal and spatial cues from the surrounding microenvironment [25].

#### 6.3.3. CD206^+^ Macrophages and Macrophage-to-Myofibroblast Transition (MMT)

In CKD, CD206^+^ macrophages accumulate at sites of tubular injury, and a subset can undergo MMT, a phenotypic shift directly engaged in interstitial fibrosis through acquiring α-smooth muscle actin (α-SMA) expression and a profibrotic phenotype [137,138,139]. MMT is regulated by a complex web of signaling involving TGF-β_1_, Smad3, Notch, JAK-STAT, PI3K/AKT, Wnt, Hedgehog, RHO/ROCK, and TNF-α pathways [23,25,141]. This switch not only highlights fibrotic remodeling but also provides a critical checkpoint in the transition from inflammation to chronic structural injury in the kidney. Significantly, Smad3 knockout models reduced CD206^+^ MMT cells and renal fibrosis and suggested that selective targeting of this pathway may have therapeutic value [23]. Targeting surface antigens such as CD206 or Smad3 and TGF-β_1_ pathways would enable precision-guided immunotherapy to suppress chronic fibrosis without impairing essential repair function.

## 7. Future Directions

Beyond their established cardiac roles, BBs act as systemic regulators by suppressing renin release and modulating downstream RAAS activity, thereby influencing vascular tone, fluid-electrolyte balance, immune signaling, and endothelial function [9,10,11,12]. These multidimensional effects highlight the need for integrative research that combines endocrine, immune, and hemodynamic readouts, supported by systems biology and omics-based approaches [142,143,144]. Clinical gaps remain. In CHF, BBs improve LVEF and survival, yet their efficacy in HFpEF and acute decompensation is variable and strongly influenced by genetic polymorphisms such as ADRB1 Arg389 and GRK5 Leu41 [15,16,89,91,92,93,94]. In CAD, BBs may modulate cytokines (e.g., IL-12p70, IL-17), but direct immunologic effects remain incompletely characterized [31]. In DN and metabolic disease, genotype-dependent responses linked to ACE I/D, eNOS, and SOD2 polymorphisms complicate drug selection, while non-vasodilating BBs can exacerbate insulin resistance and dyslipidemia. By contrast, third-generation agents (e.g., carvedilol, nebivolol) provide vasodilatory and metabolic advantages, supporting comparative studies tailored to genetic, metabolic, and inflammatory profiles [18,36,38,63,70,79,91,92,93,104,117,145]. Therapeutic innovation remains essential. Combination strategies with nonprotective agents, such as ACE inhibitors, COX-2 modulators, or IL-1β blockers, may mitigate immune and renal side effects [95,96,100,145]. Optimization of drug timing, sequencing, and duration also requires further investigation, particularly with respect to circadian RAAS activity and cytokine oscillations. Precision medicine offers the greatest potential. Pharmacogenomic markers (ADRB1, GRK5, CYP2D6, eNOS) and multi-omics data (transcriptomics, metabolomics, proteomics) integrated with machine learning (ML) can stratify patients by BB responsiveness, adverse event risk, and inflammatory phenotype [142,143,144]. Such tools may evolve into adaptive prescribing platforms that dynamically guide BB initiation, titration, and discontinuation, like current protocols for insulin or anticoagulation therapy. Finally, policy and classification frameworks must evolve. Current BB categories based on cardioselectivity or generation may be inadequate. Future classification should incorporate molecular target profiles, pharmacogenomic concordance, and disease-specific indications [146,147,148]. Vasodilating BBs may be preferred in metabolic syndrome, while ultra-selective BBs could offer safer options in patients with airway disease or cognitive impairment.

Ultimately, the transition from fixed, conventional prescribing protocols to dynamic, biomarker-guided strategies has the potential to redefine BBs as precision medicines capable of safely modulating the interconnected CV, renal, and immune systems [149,150,151,152].

## 8. Conclusions

BBs remain foundational therapies for CV diseases, including HF, MI, arrhythmias, and HTN. Their benefits arise primarily from sympathetic inhibition and renin suppression, which improve hemodynamics and reduce maladaptive remodeling. However, these same mechanisms can paradoxically produce adverse effects, such as renal hypoperfusion, pro-inflammatory immune activation, and ECG abnormalities. This risk is particularly pronounced in patients receiving “triple therapy” with BBs, RAAS inhibitors, and diuretics, where AKI incidence is elevated, underscoring the need for vigilant monitoring. The pleiotropic effects of BBs are strongly influenced by patient-specific factors, including baseline renal function, cytokine profiles, and genetic polymorphisms (e.g., ADRB1, GRK5, eNOS, CYP2D6). Future strategies should emphasize synergistic use of RAAS modulators, COX-2-targeted approaches, and selective immunotherapies (e.g., IL-1β blockade) to preserve renal function while optimizing CV remodeling.

Emerging biomarker-guided and ML platforms offer the potential to integrate pharmacogenomic and inflammatory data into individualized treatment plans. Such precision-guided approaches can help clinicians select the optimal BB type, dose, and combination for each patient, maximizing therapeutic benefit while minimizing risk.

Ultimately, BBs are not solely cardiac agents but systemic modulators of the cardio-renal-immune-endocrine axis, requiring individualized, precision-guided applications that integrate systems physiology with pharmacogenomic markers and inflammatory profiles. This approach promises to refine drug selection and dosing, advancing BBs from broad, generalized therapies to precision therapeutics.

## Figures and Tables

**Table 1:** Mechanistic Domains of BBs: Selected Studies Linking Molecular Targets to Physiologic Effects. This table summarizes key mechanistic pathways influenced by β-blockers, linking specific molecular targets (e.g., β_1_/β_2_ receptors, RAAS components, NO signaling, inflammatory cytokines) to the physiologic outcomes observed with different β-blocker classes.

Mechanistic Domain	Molecular Targets	β-Blocker Type	Effect or Outcome	References
Genetic Variants	ADRB1 Arg389Gly, GRK5, CYP2D6	Any BB (dose-adjusted)	Pharmacogenomic modifiers of BB response and metabolism	[9,10,11,12]
Sympathetic Nervous System	β_1_/β_2_-adrenergic receptors	BBs with ISA (e.g., Carteolol)	↓ HR and contractility; preserved resting tone; ↓ exercise tachycardia	[13,14]
RAAS Inhibition	Renin, Ang II, AT1R	β_1_-selective (e.g., metoprolol)	↓ Renin secretion; ↓ Ang II activity; ↓ vascular remodeling	[15,16,17]
NO Signaling Preservation	eNOS, iNOS, PGE_2_	Vasodilatory BBs (e.g., nebivolol)	↑ NO bioavailability; ↑ endothelial function	[18,19,20,21]
Fibrosis Pathway Modulation	TGF-β, Smad3, PI3K-AKT, MMT	Cardioselective BBs (e.g., bisoprolol)	↓ Cardiac and renal fibrosis; improved M1/M2 macrophage balance	[22,23]
Cytokine Modulation	TNF-α, IL-6, MCP-1	Non-selective (e.g., propranolol)	↓ Pro-inflammatory cytokine production; ↓ macrophage activation	[24,25]

Table 1 and Table 2 offer clinicians and researchers with insights into both the general and disease-spcific effects of BBs, facilitating the design of tailored treatment strategies based on molecular and clinical context. A list of abbreviations is provided at the end.

**Table 2:** BBs in Clinical Practice: Linking Therapeutic Applications to Disease-Specific Mechanisms. This table outlines major clinical conditions treated with β-blockers and links each disease state to the primary underlying mechanism, the appropriate β-blocker class, and the resulting therapeutic benefits.

Disease Context	Primary Mechanism Involved	β-Blocker Type	Clinical Outcomes and Benefits	References
Arrhythmias (AF, VT)	AV node conduction suppression	β_1_-selective or non-selective BBs	↓ Rhythm and rate disturbances; ↓ sudden cardiac death	[1,26]
Heart Failure with Reduced Ejection Fraction (HFrEF)	RAAS inhibition, SNS blockade	β_1_-selective (e.g., bisoprolol, metoprolol)	↓ Mortality; ↑ LVEF; ↓ hospitalizations	[27,28]
Hypertension	RAAS inhibition, reduced CO	β_1_-selective (e.g., atenolol); BBs with ISA (e.g., pindolol)	↓ Blood pressure; ↑ vascular compliance	[29,30,31]
Myocardial Infarction	Sympathetic modulation, anti-arrhythmic	Non-selective or β_1_-selective (e.g., atenolol, propranolol)	↓ Reinfarction; ↓ mortality; ↓ arrhythmias	[32,33,34]
Chronic Kidney Disease	RAAS inhibition, cytokine modulation	β_1_-selective (e.g., bisoprolol); vasodilatory (e.g., nebivolol)	↓ Proteinuria; ↓ CKD progression; ↑ renal protection	[31,35,36]
Thyrotoxicosis	β_1_-, β_2_-receptor blockade	Non-selective (e.g., propranolol)	↓ Tremor and palpitations; ↓ T_4_→T_3_ conversion	[37,38]

Table 1 and Table 2 offer clinicians and researchers with insights into both the general and disease-specific effects of BBs, facilitating the design of tailored treatment strategies based on molecular and clinical context.

**Table 3:** Hormonal and Metabolic Effects of Long-term BB Therapy. This table summarizes the key hormonal and metabolic alterations associated with chronic BB use and offers a practical framework for clinical application.

Authors (Year)	Effect of BB Therapy	Clinical Insight/Implication
Gullestad et al. (1989) [65]	Modulates neurohormonal responses (cortisol, epinephrine, prolactin) during physical stress	BBs blunt stress hormone surges during exertion; may limit physiologic adaptation
DeMorrow (2018) [64]	Describes HPA axis physiology and BBs influence on stress adaptation	Chronic HPA modulation by BBs may alter endocrine resilience and stress response
Burford et al. (2017) [66]	Alters glucocorticoid signaling via HPA axis and cardiovascular effects	Prolonged BBs use may increase CV risk by disrupting glucocorticoid homeostasis
Bugajski et al. (1995) [67]	Shows β-adrenergic stimulation of HPA axis under stress	BBs reduce stress-induced cortisol secretion; consider stress-sensitive disorders
Macdonald et al. (1984) [30]	Inhibits lactate and free fatty acid mobilization during exercise	BBs alter exercise metabolism; caution in cardiac patients requiring energy reserve
Oki et al. (2009) [68]	Propranolol normalizes cortisol in AIMAH (adrenal incidentaloma)	May be therapeutic in subclinical Cushing’s syndrome via β-receptor modulation
Wiersinga (1991) [37]	Inhibits type 1 deiodinase, lowering T_3_ levels	Effective in hyperthyroidism; monitor for hypothyroid risk with long-term use
Geffner & Hershman (1992) [38]	Reduces adrenergic symptoms in thyrotoxicosis	BBs alleviate hyperthyroid symptoms; recommend thyroid function monitoring
McGill (2009) [69]	Non-selective BBs impair insulin sensitivity in diabetes	Avoid non-selective BBs in diabetics; consider vasodilatory agents
Kveiborg et al. (2010) [70]	Carvedilol preserves, metoprolol impairs insulin-stimulated endothelial function	Choose carvedilol over metoprolol for improved metabolic safety in diabetics
Zanchetti et al. (1983) [71]	Suppresses plasma renin activity	Monitor RAAS suppression; adjust antihypertensive regimen accordingly
Knox et al. (1980) [72]	Alters renal sodium balance under mineralocorticoid influence	Monitor fluid/electrolyte status in long-term BBs therapy for volume-sensitive patients

**Table 4:** Mechanisms of β_1_-Selective BBs in Cardiac and Renal Physiology. This table summarizes how β_1_-selective BBs regulate key cardiac and renal functions.

Citation	Study Type	BB Type	Mechanism of Action	Target System	Physiologic/Clinical Effect	Example Drugs	Model
Wang et al. (2006) [7]	Experimental	β_1_-stimulation (untreated)	Preserves renal afferent arterioles from NE-induced oxidative stress via cAMP	Renal	Protective against oxidative injury	N/A	Animal (Rabbit)
Bruck et al. (2004) [13]	Clinical (in vivo)	BBs with ISA	Partial β_2_ agonism → mild vasodilation & ↑ HR	Cardiac & vascular	↓ HR/bradycardia risk; preserved exercise tolerance	Carteolol, Alprenolol	Human
Nyberg et al. (1979) [14]	Clinical (pharmacology)	BBs with ISA	Partial β_2_ agonism → mild vasodilation & ↑ HR	Cardiac & vascular	↓ HR/bradycardia risk; preserved exercise tolerance	Penbutolol	Human
Cruickshank et al. (2010) [45]	Review (HF/ISA)	BBs with ISA	Partial β_2_ agonism → mild vasodilation & ↑ HR	Cardiac & vascular	↓ HR/bradycardia risk; preserved exercise tolerance	Carteolol, Alprenolol	Human
Khan et al. (2023) [74]	Clinical review	β_1_-selective BBs	Inhibit β_1_-adrenergic stimulation → ↓ HR, ↓ contractility	Cardiac	↓ Myocardial O_2_ demand; ↑ diastolic filling; anti-ischemic	Metoprolol, Bisoprolol	Human
Khan et al. (2023) [74]	Clinical review	Vasodilatory BBs	α_1_-blockade → ↓ PVR & vasodilation	Vascular	↓ Afterload; ↑ CO	Carvedilol, Labetalol	Human
Khan et al. (2023) [74]	Clinical review	Vasodilatory BBs	↑ NO bioactivity → ↓ PVR	Vascular	↓ BP; ↑ CO	Nebivolol	Human
Prijic et al. (2014) [75]	Clinical review	BBs (general)	↓ SNS overdrive → ↓ afterload; reverse remodeling; ↓ arrhythmias	Cardiac	↓ Angina; ↓ HF symptoms; ↑ survival	Carvedilol, Metoprolol	Human
Wołowiec et al. (2023) [1]	Review (clinical)	Non-selective BBs	Block β_1_ & β_2_ → ↓ HR & ↓ renin (possible ↑ vasoconstriction)	Cardiorenal	Mixed BP effects; caution in asthma	Propranolol, Nadolol	Human
Baltogiannis et al. (2019) [26]	Brief review	Non-selective BBs	Block β_1_ & β_2_ → ↓ HR & ↓ renin (possible ↑ vasoconstriction)	Cardiorenal	Mixed BP effects; caution in asthma	Propranolol, Nadolol	Human
Han et al. (2020) [76]	Network meta-analysis	Non-selective BBs	Block β_1_ & β_2_ → ↓ HR & ↓ renin (possible ↑ vasoconstriction)	Cardiorenal	Mixed BP effects; caution in asthma	Propranolol, Nadolol	Human
Han et al. (2020) [76]	Network meta-analysis	Nadolol (non-selective)	↓ SNS effect on heart → ↓ QT dispersion	Cardiac electrical	↓ Risk of sudden death in LQTS	Nadolol	Human
Schweda et al. (2007) [15]	Review (renal physiology)	β_1_-selective BBs	Inhibit β_1_ on JG cells → ↓cAMP → ↓ renin	Renal	↓ RAAS activation; ↓ BP	Atenolol, Metoprolol	Animal & Human
Kurtz et al. (2012) [77]	Review (renal physiology)	β_1_-selective BBs	Inhibit β_1_ on JG cells → ↓ cAMP → ↓ renin	Renal	↓ RAAS activation; ↓ BP	Atenolol, Metoprolol	Animal & Human
Kurtz et al. (2011) [16]	Review (renal physiology)	β_1_-selective BBs	Inhibit β_1_ on JG cells → ↓ cAMP → ↓ renin	Renal	↓ RAAS activation; ↓ BP	Atenolol, Metoprolol	Animal & Human
Manis et al. (2019) [78]	Experimental physiology	BBs (general)	↓ Renin, ↓ Na^+^ reabsorption, ↑ renal O_2_ balance	Renal	Stabilizes BP; ↑ renal tissue oxygenation	Atenolol, Metoprolol, Propranolol	Animal & Human
Kumar et al. (2023) [79]	Review (diabetes/renal)	BBs (general)	↓ Renin, ↓ Na^+^ reabsorption, ↑ renal O_2_ balance	Renal	Stabilizes BP; ↑ renal tissue oxygenation	Carvedilol, Nebivolol, Bisoprolol	Human
Hall et al. (2016) [59]	Observational study	BBs (general)	↓ Renin, ↓ Na^+^ reabsorption, ↑ renal O_2_ balance	Renal	Stabilizes BP; ↑ renal tissue oxygenation	Metoprolol, Bisoprolol, Carvedilol	Human
Strauss et al. (2023) [80]	Clinical review	BBs (general)	↓ Renin, ↓ Na^+^ reabsorption, ↑ renal O_2_ balance	Renal	Stabilizes BP; ↑ renal tissue oxygenation	Atenolol, Metoprolol, Nebivolol	Human

**Table 5:** Downstream Molecular Pathways Modulated by β-Blockers. This table illustrates how BBs modulate key downstream pathways, including inhibition of the RAAS pathway, regulation of COX-2 expression, modulation of PGE_2_–iNOS–NO signaling, and suppression of aldosterone activity. Notably, PGE_2_ can paradoxically inhibit iNOS and NO production in immune cells, which may contribute to altered immune responses during chronic inflammation.

BBs	RAAS	COX-2	PGE_2_ and iNOS/NO Pathway	NO	Aldosterone
Block β_1_-adrenergic receptors on juxtaglomerular cells, reducing renin release and suppressing RAAS activity.	Under normal conditions, RAAS activation leads to: ANG II–mediated COX-2 upregulation. Aldosterone secretion via adrenal cortex stimulation. Vasopressin (ADH) release via hypothalamic–pituitary axis. β-blockers attenuate these downstream effects.	ANG II stimulates COX-2 expression in vascular and renal cells. COX-2 catalyzes arachidonic acid conversion into prostaglandins, including PGE_2_, which regulate inflammation and vascular tone.	COX-2–derived PGE_2_ may inhibit iNOS expression via EP2/EP4 receptors, leading to decreased NO synthesis. NO acts as a vasodilator and modulates immune cell differentiation.	NO mediates: Vasodilation, lowering blood pressure. Anti-inflammatory effects. Maintenance of renal blood flow and function. Reduced NO contributes to hypertension, inflammation, and renal dysfunction.	Aldosterone binds mineralocorticoid receptors, promoting sodium and water retention, affecting renal function through volume status and elevating blood pressure.

## Data Availability

No new data were created or analyzed in this study. Data sharing does not apply to this article.

## Abbreviations

The following abbreviations are used in this manuscript:

Abbreviation	Full Term
ACE	Angiotensin-Converting Enzyme
ACS	Acute Coronary Syndrome
ACTH	Adrenocorticotropic Hormone
ADHF	Acute Decompensated Heart Failure
ADRB1	Beta-1 Adrenergic Receptor
ADRB2	Beta-2 Adrenergic Receptor
AIMAH	ACTH-Independent Macronodular Adrenal Hyperplasia
AKI	Acute Kidney Injury
AMPK	AMP-Activated Protein Kinase
ANP	Atrial Natriuretic Peptide
ANG II	Angiotensin II
APOE	Apolipoprotein E
AT1R	Angiotensin II Type 1 Receptor
AT2R	Angiotensin II Type 2 Receptor
BBs	Beta-Blockers
BNP	B-Type Natriuretic Peptide
BP	Blood Pressure
CAD	Coronary Artery Disease
CD206	Mannose Receptor (Marker of M2 Macrophages)
CHF	Congestive Heart Failure
CKD	Chronic Kidney Disease
COL4A1	Collagen Type IV Alpha 1 Chain
CO	Cardiac Output
COPD	Chronic Obstructive Pulmonary Disease
COX-2	Cyclooxygenase-2
CPVT	Catecholaminergic Polymorphic Ventricular Tachycardia
CRH	Corticotropin-Releasing Hormone
CRP	C-Reactive Protein
CNS	Central Nervous System
CV	Cardiovascular
CYP2D6	Cytochrome P450 2D6
DCM	Diabetic Cardiomyopathy
DN	Diabetic Nephropathy
ECG	Electrocardiogram
ECM	Extracellular Matrix
eNOS	Endothelial Nitric Oxide Synthase
FEV_1_	Forced Expiratory Volume in 1 s
FFA	Free Fatty Acids
FVC	Forced Vital Capacity
GAL3	Galectin-3
GFR	Glomerular Filtration Rate
GLUT1/4	Glucose Transporter 1/4
GRK5	G Protein-Coupled Receptor Kinase 5
HDL	High-Density Lipoprotein
HF	Heart Failure
HFmrEF	Heart Failure with Mildly Reduced Ejection Fraction
HFpEF	Heart Failure with Preserved Ejection Fraction
HFrEF	Heart Failure with Reduced Ejection Fraction
HR	Heart Rate
HPA axis	Hypothalamic–Pituitary–Adrenal Axis
IL-1β	Interleukin-1 Beta
IL-4	Interleukin-4
IL-6	Interleukin-6
IL-10	Interleukin-10
IL-12p70	Interleukin-12 subunit p70
IL-13	Interleukin-13
IL-17	Interleukin-17
iNOS	Inducible Nitric Oxide Synthase
IRF1	Interferon Regulatory Factor 1
ISA	Intrinsic Sympathomimetic Activity
JAK	Janus Kinase
LDL	Low-Density Lipoprotein
LQTS	Long QT Syndrome
LVEF	Left Ventricular Ejection Fraction
MCP-1	Monocyte Chemoattractant Protein-1
MI	Myocardial Infarction
ML	Machine Learning
MIP-1β	Macrophage Inflammatory Protein-1 Beta
M1	Classically Activated (Pro-inflammatory) Macrophages
M2	Alternatively Activated (Anti-inflammatory/Reparative) Macrophages
MMP-2	Matrix Metalloproteinase-2
MMT	Macrophage-to-Myofibroblast Transition
MOD	Myocardial Oxygen Demand
NF-κB	Nuclear Factor kappa-light-chain-enhancer of activated B cells
NLRP3	NOD-, LRR- and pyrin domain-containing protein 3
NO	Nitric Oxide
NOS	Nitric Oxide Synthase
nNOS	Neuronal Nitric Oxide Synthase
PGE_2_	Prostaglandin E_2_
PI3K	Phosphoinositide 3-Kinase
PKC	Protein Kinase C
PLA2	Phospholipase A2
PM	Poor Metabolizers
PPARγ	Peroxisome Proliferator-Activated Receptor Gamma
PVR	Peripheral Vascular Resistance
RAAS	Renin–Angiotensin–Aldosterone System
RBF	Renal Blood Flow
RCT	Randomized Controlled Trial
ROS	Reactive Oxygen Species
SCD	Sudden Cardiac Death
Smad3	Mothers Against Decapentaplegic Homolog 3
SOD2	Superoxide Dismutase 2
SNS	Sympathetic Nervous System
STAT1	Signal Transducer and Activator of Transcription 1
T_3_	Triiodothyronine
T_4_	Thyroxine
TGF-β_1_	Transforming Growth Factor Beta 1
TIMP-2	Tissue Inhibitor of Metalloproteinases-2
TNF-α	Tumor Necrosis Factor Alpha
UM	Ultra-rapid Metabolizers
α-SMA	Alpha-Smooth Muscle Actin
